# Detection of Protein Complexes Based on Penalized Matrix Decomposition in a Sparse Protein–Protein Interaction Network

**DOI:** 10.3390/molecules23061460

**Published:** 2018-06-15

**Authors:** Buwen Cao, Shuguang Deng, Hua Qin, Pingjian Ding, Shaopeng Chen, Guanghui Li

**Affiliations:** 1 College of Information and Electronic Engineering, Hunan City University, Yiyang 413000, China; qinhua_hcu@163.com; 2 College of Computer Science and Electronic Engineering, Hunan University, Changsha 410082, China; dpj@hnu.edu.cn (P.D.); ghli16@163.com (G.L.); 3 College of Mathematics and Computer Science, Hunan Normal University, Changsha 410081, China; chenshaopeng2010@gmail.com; 4 School of Information Engineering, East China Jiaotong University, Nanchang 330013, China

**Keywords:** protein–protein interaction (PPI), clustering, protein complex, penalized matrix decomposition

## Abstract

High-throughput technology has generated large-scale protein interaction data, which is crucial in our understanding of biological organisms. Many complex identification algorithms have been developed to determine protein complexes. However, these methods are only suitable for dense protein interaction networks, because their capabilities decrease rapidly when applied to sparse protein–protein interaction (PPI) networks. In this study, based on penalized matrix decomposition (*PMD*), a novel method of penalized matrix decomposition for the identification of protein complexes (i.e., PMD_pc_) was developed to detect protein complexes in the human protein interaction network. This method mainly consists of three steps. First, the adjacent matrix of the protein interaction network is normalized. Second, the normalized matrix is decomposed into three factor matrices. The PMD_pc_ method can detect protein complexes in sparse PPI networks by imposing appropriate constraints on factor matrices. Finally, the results of our method are compared with those of other methods in human PPI network. Experimental results show that our method can not only outperform classical algorithms, such as CFinder, ClusterONE, RRW, HC-PIN, and PCE-FR, but can also achieve an ideal overall performance in terms of a composite score consisting of F-measure, accuracy (ACC), and the maximum matching ratio (MMR).

## 1. Introduction

The identification of protein complexes is highly beneficial for the investigation of all kinds of organisms to understand biological processes and determine inherent organizational structures within cells [1]. The dramatic development of computational methods stimulates many protein complex identification algorithms for protein–protein interaction (PPI) networks, which are generally organized into three catalogs. The first catalog includes clustering methods that are also divided into three sub-catalogs. First, the local search approaches based on density are used to identify densely connected subgraphs in PPI networks, in which subgraphs with density above a pre-defined threshold, such as MCODE (Molecular Complex Detection) [2], CFinder (a software tool for network cluster detection) [3], DPCLus (a Density-Periphery based graph CLustering software) [4], and ICPM (Iterative Clique Percolation Method) [5], are considered protein complexes. However, these approaches tend to neglect surrounding proteins that are connected to the kernel clusters with sparse links, which can show experimentally validated true interactions [6]. Another kind of method for detecting protein complexes uses classical hierarchy clustering techniques, which mainly depend on the distance between proteins to detect meaningful groups [6] and contain HC-PIN ((fast Hierarchical Clustering algorithm for Protein Interaction Network, agglomerative method) [7] and G-N algorithms (divisive method) [8]. Many hierarchical clustering methods employ similarities among the proteins that are calculated on the basis of network topology characteristics or biological meaning due to the further development of clustering technology. Such approaches mainly include NEMO (NEtwork MOdule identification) [9], ClusterONE (Clustering algorithm with Overlapping Neighborhood Expansion) [10], RFC (Rough Fuzzy Clustering) [11], MINE (Module Identification in Networks) [12], PageRankNibble [13], SPICi (Speed and Performance In Clustering,) [14], PCE-FR (Pseudo-Clique Extension based on Fuzzy Relation) [15], MTGO (Module detection via Topological information and GO knowledge) [16], WCOACH (Weighted COACH) [17], DCAFP (Density-based Clustering Approach for identifying overlapping protein complexes with Functional Preferences) [18], and cwMINE (Combined Weight of Module Identification in Networks) [19]. Experimental results show that these novel methods greatly outperform classical hierarchical clustering approaches. Except for the aforementioned clustering approaches, many other protein complex detection algorithms, such as RNSC (Restricted Neighborhood Search Clustering) [20], MCL [1], RRW (Repeated Random Walks algorithm) [21], CMC (Clustering-based on Maximal Cliques) [22], Coach [23], and AP (Affinity Propagation) with its variant [24] have achieved satisfactory results.

Another type of method used to detect protein complexes employs an intelligent optimization algorithm, which seeks the optimal solution of PPI based on a heuristic concept [25]. For large databases, the complexity of intelligent optimization algorithms is too high to run a correct consequence. The major weakness of the aforementioned methods is that their performance deteriorates when they are employed to sparse PPI networks [19,26]. To address this problem, matrix decomposition is proposed to improve the disadvantages of these methods. A co-clustering algorithm based on the adjacent matrix of PPI networks was proposed [6] and obtained overlapping and non-overlapping protein complexes successfully. The results show that the method reached a remarkable balance between network coverage and accuracy (ACC) and outperformed classical methods. Matrix factorization can be mainly organized into two main levels. The first level is the non-negative matrix factorization (NMF) (which integrates gene ontology (GO), gene expression data, and the PPI network to form the corresponding adjacency matrix and then decomposes it with common factors to achieve the overlapping functional modules with high ACC [27]). Zhang et al. [28] proposed sparse network-regularized multiple NMFs (SNMNMFs) to identify the microRNA regulatory modules and demonstrated the ideal performance of the proposed method in ovarian cancer dataset. The second level is the penalized matrix decomposition (PMD), which is widely applied in various datasets, such as microarray data [29], including gene expression data, and proteomic datasets [30].

Inspired by Ref. [24], PMD_pc_, an approach used to identify the protein interaction network of protein complexes was originally proposed. First, the adjacent matrix of the protein interaction network was normalized. Second, the normalized matrix was decomposed into three factor matrices. Finally, the PMD_pc_ algorithm and several classical algorithms were executed from the well-investigated human PPI network. The experimental results show that our approach achieved satisfactory performance in terms of F-measure, ACC, and maximum matching ratio (MMR).

## 2. Results and Discussion

When PMD_pc_ is applied to identify the protein complexes in PPI network, the parameters of $c_{1}$, $c_{2}$, and k are crucial for the decomposition of the network. Considering that u should be sparse, we take $c_{1}=0.25\times \sqrt{n}$ and $c_{2}=0.25\times \sqrt{p}$ [31].

**Figure 1:**
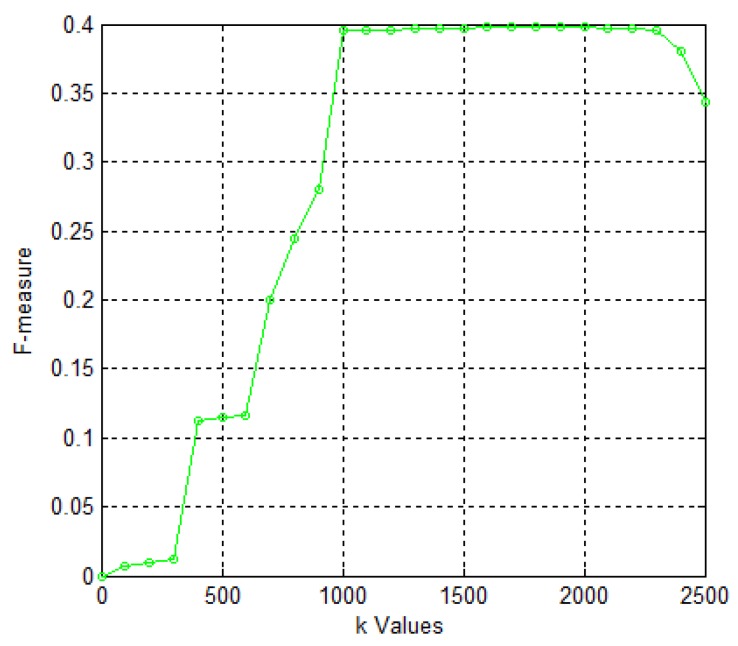
Values of F-measure for different values of *k* ∈ (0, 2500] with a 100 increment in HPRD dataset.

To study the parameter of *k* on the effect on the experimental results, we repeated the execution of algorithm and studied how the algorithm behaves in terms of F-measure and let k∈(0,2500] with a 100 increment. The detailed experimental results with different k values are presented in Figure 1. From Figure 1, we can clearly see that k is less than 1000; the experimental results fall short of satisfaction.

The value of the F-measure increases gradually until k=1600 with the increase in k, such that the maximum value of 0.398, the F-measure, displays a steady state when it changes from 1600 to 2000. When k is greater than 2000, the value of F-measure shows a downward trend. Therefore, k is set to 2000.

Five classical protein complex algorithms, namely, CFinder [3], ClusterONE [10], RRW [21], HC-PIN [7], and PCE-FR [15], are applied on human PPI network of HPRD (Human Protein Reference Database, HPRD) to demonstrate the performance of $PMD_{pc}$. The complexes of the aforementioned algorithms with sizes less than 2 are filtered in our work. Moreover, the parameters of each method that is compared with our method are set using the default values recommended by the authors. The experimental result is shown in Table 1.

Table 1 shows that $PMD_{pc}$ achieves a satisfactory performance on human PPI networks. Particularly, $PMD_{pc}$ obtains the highest value of recall, F-measure, ACC, and Sep, which are 0.356, 0.398, 0.362, and 0.777, respectively. These results are significantly superior to the five other algorithms. Furthermore, CFinder achieves the highest precision of 0.959 and the lowest MMR of 0.017. ClusterONE identifies 755 protein complexes and achieves the highest MMR of 0.084. These values elaborate that our approach achieved an ideal result in identifying protein complexes from sparse PPI networks.

From Table 1, we can also clearly see that our method obtains the second highest value of MCC, which is 12.28% lower than that of ClusterONE. It demonstrates that our method achieved satisfactory performance in dealing imbalanced data.

**Figure 2:**
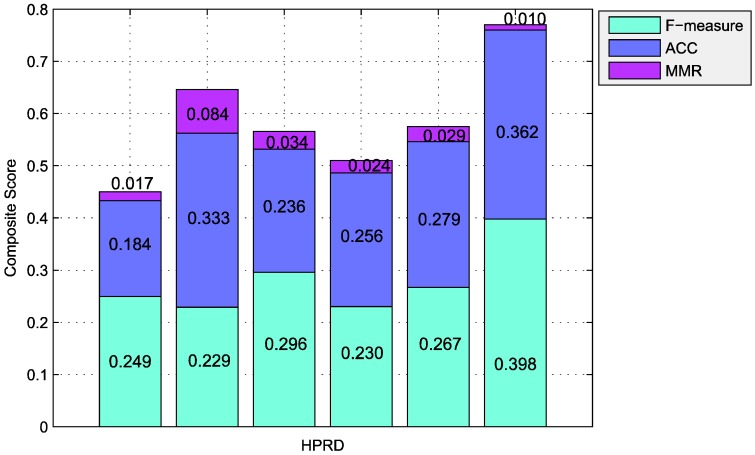
Results comparison of the six algorithms in HPRD dataset using CHPC2012 gold standard dataset. Columns correspond to the following algorithms, CFinder, ClusterONE, HC-PIN, PCE-FR, and $PMD_{pc}$ from left to right. Various color of the same columns denotes the individual components of the composite score of the algorithm (cyan = F-measure, blue = ACC, and purple = MMR). The total height of each column is the value of the composite score for a special algorithm in a special dataset. Large score shows the clustering result is better.

To void the advantage of some evaluation metric, the composite score [24] is employed to wrap up the global performance. Interestingly, the composite comparison of our method shows absolute advantage in terms of F-measure, accuracy, and maximum matching ratio. Figure 2 presents the comparison results of the six algorithms on the HPRD dataset. The composite score of F-measure, accuracy, and maximum matching ratio is 0.770, which is 19.20% higher than the highest value of the five other methods. It further demonstrates the effectiveness of our method.

## 3. Materials and Methods

### 3.1. Materials and Datasets

Our method is applied to detect the protein complexes in the human PPI dataset downloaded from Ref. [24], in which 9459 proteins and 36,935 interactions with the density of 0.0008 are included. The gold standard dataset is employed to evaluate the performance of the protein complexes identified in sparse PPI networks, which is CHPC2012 [32], integrating three databases, namely, CORUM [33], HPRD [34], and PINdb [35], and includes 1389 complexes and 3065 proteins.

### 3.2. Methods

Consider a sample dataset that consists of p eigenvectors in n samples, which is described by a matrix X with size n×p [30]. Without loss of generality, we assume that the means of column and row X are zero. The singular value decomposition of matrix X can be written as follows:

(1) $$X=U\Delta V^{T},U^{T}U=I_{n},V^{T}V=I_{p}$$

The decomposition of sparse matrix is executed by imposing additional constraints on U and V. The single-factor PMD can be optimized using the following objective function, which is formulated as [30]

(2) $$\begin{array}{l} \arg\underset{\delta ,u,v}{\min}\frac{1}{2}||\eta -\delta uv^{T}|{|}_{F}^{2},\\ s.t.||u|{|}_{2}^{2}=1,||v|{|}_{2}^{2}=1,\\ P_{1}\left(u\right)\leq c_{1},P_{2}\left(v\right)\leq c_{2},\delta \geq 0. \end{array}$$

in which u is a column of U, v is a column of V, δ is a diagonal element of the matrix of η, ${\|•\|}_{F}$ is the Frobenius norm, and $P_{1}$ and $P_{2}$ are penalty functions that have variety of forms [30].

Let U and V be n×R and p×R orthogonal matrices, respectively, and Δ a diagonal matrix with diagonal elements $\delta_{r}$ [30]

(3) $$\frac{1}{2}{\left\|\eta -U\Delta V^{T}\right\|}_{F}^{2}=\frac{1}{2}{\left\|\eta \right\|}_{F}^{2}-\sum_{r=1}^{R}u_{r}^{T}\eta v_{r}\delta_{r}+\frac{1}{2}\sum_{r=1}^{R}\delta_{r}^{2}$$

Therefore, when R=1, we can infer that u and v satisfy Equation (7) and the following condition:

(4) $$\begin{array}{l} \arg\underset{u,v}{\max u^{T}}\eta v\\ s.t.\;{\left\|u\right\|}_{2}^{2}=1,\;{\left\|v\right\|}_{2}^{2}=1,\;P_{1}\left(u\right)\leq c_{1},\;P_{2}\left(v\right)\leq c_{1} \end{array}$$

Moreover, δ satisfies Equation (2) when $\delta =u^{T}\eta v$.

The optimization problem in Equation (4) can be applied to the following biconvex optimization [30]:

(5) $$\begin{array}{l} \arg\underset{u,v}{\max}u^{T}\delta v\\ s.t.\;{\left\|u\right\|}_{2}^{2}\leq 1,\;{\left\|v\right\|}_{2}^{2}\leq 1,\;P_{1}\left(u\right)\leq c_{1},\;P_{2}\left(v\right)\leq c_{2} \end{array}$$

Equation (5) satisfies Equation (4) based on the appropriate value of c [30]. Equation (5) is called the single factor PMD, and the iterative algorithm used to optimize it is described in Algorithm 1:

**Algorithm 1.** Calculating the single factor of PMD.**Step1.** Initialize v and let unit $L_{2}-norm$. **Step2.** Interate until convergence: (i)$u\leftarrow \arg\;\max_{u}\;u^{T}\delta v,\;s.t.{\left\|u\right\|}_{2}^{2}\leq 1,P_{1}\left(u\right)\leq c_{1}$(ii)$v\leftarrow \arg\;\max_{v}\;u^{T}\delta v,\;s.t.{\left\|v\right\|}_{2}^{2}\leq 1,P_{2}\left(v\right)\leq c_{2}$ **Step3.**
$d\leftarrow u^{T}\delta v$


Equation (2) is computed repeatedly to obtain other PMD factors. The corresponding algorithm is described in Algorithm 2.

**Algorithm 2.** Calculating the k factor of PMD.**Step1.**$\eta^{1}\leftarrow \eta$; **Step2.** For r∈1,2,…,R(i)The single factor PMD (Algorithm 1) is executed on the matrix of $\eta^{r}$, computing $u_{r},v_{r},\delta_{r}$, respectively;(ii)$\eta^{r+1}\leftarrow \eta^{r}-\delta_{r}u_{r}v_{r}^{T}$

The constraint is imposed on u and v with $L_{1}-norm$, i.e., ${\left\|u\right\|}_{1}\leq c_{1},{\left\|v\right\|}_{1}\leq c_{2}$. By selecting parameters $c_{1}$ and $c_{2}$ appropriately, PMD can make factors u and v sparse. Generally, $c_{1}$ and $c_{2}$ should be restricted to ranges $1\leq c_{1}\leq \sqrt{n}$ and $1\leq c_{2}\leq \sqrt{p}$. Thus, the PMD method is shaped as $PMD(L_{1},L_{2})$, which is described as follows:

(6) $$\begin{array}{l} \arg\underset{u,v}{\max}u^{T}\eta v\\ s.t.\;{\left\|u\right\|}_{2}^{2}\leq 1,\;{\left\|v\right\|}_{2}^{2}\leq 1,\;{\left\|u\right\|}_{1}\leq c_{1},{\left\|v\right\|}_{1}\leq c_{2} \end{array}$$

Let S denote the operator of the soft threshold, i.e., $S\left(a,c\right)=\mathrm{sgn}\left(a\right){\left(\left|a\right|-c\right)}_{+}$, in which c>0, $x_{+}=\left\{ \begin{array}{ll} x & x>0\\ 0 & x\leq 0 \end{array}\right.$. The corresponding theorem is as follows:

**Theorem 1.**  *Considering the optimization problem*

(7) $$\begin{array}{l} \arg\underset{u}{\max}u^{T}a\\ s.t.\;{\left\|u\right\|}_{2}^{2}\leq 1,\;{\left\|u\right\|}_{1}\leq c. \end{array}$$

The solution is $u=\frac{S(a,\Delta )}{{\left\|S(a,\Delta )\right\|}_{2}}$. If ${\left\|u\right\|}_{1}\leq c$, then Δ = 0; otherwise, ${\left\|u\right\|}_{1}=\;c$ *s.t.* Δ > 0. The detailed proof regarding the theorem can be found in Ref. [30]. The analysis shows the solution of Equation (6) with Algorithm 1. According to Theorem 1, the single factor PMD can be optimized, as shown in Algorithm 3:

**Algorithm 3.** The optimization process of the single factor PMD.**Step1.** Initialize v and let unit $L_{2}-norm$. **Step2.** Iterate until convergence:
(i)$u\leftarrow \frac{S(Xv,\Delta_{1})}{{\left\|S(Xv,\Delta_{1})\right\|}_{2}}$, if ${\left\|u\right\|}_{1}\leq c_{1}$, then $\Delta_{1}$ = 0, else ${\left\|u\right\|}_{1}=\;c_{1},s.t.$, $\Delta_{1}$ > 0(ii)$u\leftarrow \frac{S(X^{T}u,\Delta_{2})}{{\left\|S(X^{T}u,\Delta_{2})\right\|}_{2}}$, if ${\left\|v\right\|}_{1}\leq c_{2}$, then $\Delta_{2}$ = 0, else ${\left\|v\right\|}_{1}=c_{2},s.t.,$ $\Delta_{2}$ > 0 **Step3.**
$d\leftarrow u^{T}\delta v$

To obtain the sparse factors of u and v, we let $c_{1}=c\sqrt{n},c_{2}=c\sqrt{p}$, and the values of $\Delta_{1}$ and $\Delta_{2}$ are selected by the binary search.

For comprehensive discussion, discovered protein complexes and gold standard dataset are matched. The following evaluation measures are employed in this study.

F-measure. Two protein complexes, namely, p and g, are generated from the predicted protein complex and gold standard sets, respectively. The overlapping score os(p,g) quantizes the closeness between the sets and is defined as follows [24]:

(8) $$\mathrm{os}(p,g)=\frac{\left|C_{p}\cap C_{g}\right|}{\left|C_{p}|•|C_{g}\right|}$$

in which $C_{p}$, $C_{g}$ denote protein complex sets p and g, respectively. If os(p,g)≥θ, then the two complexes are matched, in which θ is the threshold. θ is set as 0.2, which is consistent with many experiments for protein complex identification [24]. Let P and G represent the detected protein complex and gold standard sets, respectively; $N_{cp}$ describes the number of identified protein complexes that match at least one gold standard set, i.e., $N_{cp}=|\{p|p\in P,\exists g\in G,os(p,g)\geq \theta \}|$; and $N_{cp}$ presents the number of gold standard protein complexes that match at least one identified complex, that is $N_{cg}=|\{g|g\in G,\exists p\in P,os(p,g)\geq \theta \}|$. F-measure is mathematically defined as [24]

(9) $$F-measure=\frac{2\times Precision\times Recall}{Precision+Recall}$$

in which $Precision=N_{cp}/|P|,\;Recall=N_{cg}/|G|$. F-measure is defined as the harmonic mean of precision and recall, which can evaluate the overall performance of the detection methods.

ACC (Accuracy, ACC). ACC is used to quantify the quality of detected protein complexes, which is the geometric means of sensitivity and positive predictive value, PPV. The corresponding formulas are described as follows [24]:

(10) $$ACC=\sqrt{S_{n}\times PPV}$$

in which $S_{n}=\frac{\sum_{i=1}^{n}\max_{j=1}^{m}t_{ij}}{\sum_{i=1}^{n}n_{i}}$, $PPV=\frac{\sum_{j=1}^{m}\max_{i=1}^{n}t_{ij}}{\sum_{j=1}^{m}\sum_{i=1}^{n}t_{ij}}$.

Sep (Separation, Sep). To void the case wherein proteins of a gold standard complex are matched with several identified protein complexes, Sep is used to measure the one-to-one correspondence between generated protein complexes and gold standard protein complexes. The formula is described as follows [24]:

(11) $${\mathrm{Sep}}_{g}=\frac{\sum_{i=1}^{n}\sum_{j=1}^{m}Sep_{ij}}{n},\;{\mathrm{Sep}}_{p}=\frac{\sum_{j=1}^{m}\sum_{i=1}^{n}Sep_{ij}}{m},\mathrm{Sep}=\sqrt{{\mathrm{Sep}}_{g}\times Sep_{p}},$$

in which ${\mathrm{Sep}}_{ij}=\frac{{\left(t_{ij}\right)}^{2}}{\sum_{i=1}^{n}t_{ij}*\sum_{j=1}^{m}t_{ij}}$. In Formulas (10) and (11), n is the number of protein complexes in the gold standard dataset, m is the number of identified protein complexes, $t_{ij}$ denotes the size of intersection between the ith gold standard complex and the jth detected complex, and $n_{i}$ denotes the number of proteins included in the ith gold standard complex.

MMR (Maximum Matching Ratio). MMR is used to describe the maximum one-to-one matching between the identified and gold standard protein complexes, which are defined as follows [24]:

(12) $$MMR(g,p)=\frac{\sum_{i=1}^{n}\max_{j=1}^{m}os(g_{i},p_{j})}{N_{i}}$$

in which os represents the overlapping score between two protein complexes, $g_{i}$ is the ith gold standard complex, and $p_{j}$ represents the jth identified protein complex.

MCC (Matthews Correlation Coefficient). MCC is widely used in bioinformatics as a performance metric that can handle imbalanced data. The formula is described as follows [24]:

(13) $$MCC=\frac{TP\times TN-FP\times FN}{\sqrt{\left(TP+FN)(TP+FP)(TN+FP)(TN+FN\right)}}$$

in which TP, TN, FP, and FN mean the true positive, true negative, false positive, and false negative, respectively.

### 3.3. Detection of Protein Complexes Using $PMD_{pc}$

A PPI network is usually modeled as an undirected weight graph G=(V,E,ω), in which V represents a set of nodes (proteins), *E* is a set of edges (protein pairs), and ω is a set of similarity value between each protein pairs. The similarity of GO (Gene Ontology, GO) terms is mathematically expressed as follows [36]:

(14) $$Sim(i,j)=\frac{|N(i)\cap N(j)|}{\min(N(i),N(j))}$$

in which Sim(i,j) indicates the GO similarity of the protein pair (i,j). N(i) denotes the number of GO terms that annotate the protein i. The PPI network is stocked as the matrix X with a size of n×n, which is transformed into the vertex–PCA matrix X of size n×p by the principal component analysis, in which each row of X represents a protein in all n samples (protein complexes), and each column of X represents the expression level of a sample in all p proteins.

**Figure 3:**
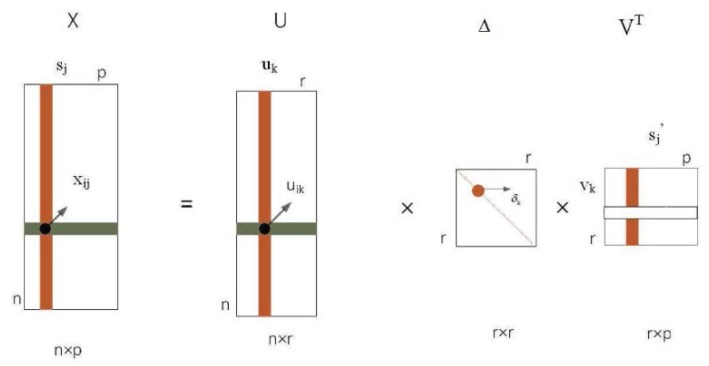
Graphical description of $PMD_{pc}$. Matrix X is decomposed into two base matrices, namely, *U*, *V*, and a diagonal matrix Δ.

According to Section 3.2, the matrix X is decomposed into three matrices, namely, U,V, and Δ by PMD. The graphical description of $PMD_{pc}$ is shown in Figure 3, in which $u_{k}$ is the kth principal component, $v_{k}$ is the kth expression model of the principal component, and $u_{ik}$ indicates that the kth protein is projected on the kth protein complex. Therefore, matrix U is decomposed into several clusters (protein complexes) due to matrix decomposition.

$PMD_{pc}$ is implemented in Java, and all experiments are performed on an Intel(R) Core(TM) i7-5557U CPU with 2.2 GHz and 8 GB RAM running Windows 7.0. The elapsed time is 9533 s.

## 4. Conclusions

The identification of protein complex helps us to discover and understand the cellular organizations and biological functions in PPI networks. Previous computational approaches mainly identified protein complexes in dense PPI networks, which had inferior performances in sparse PPI networks. In this work, $PMD_{pc}$ is proposed on the basis of the penalized matrix decomposition to detect protein complexes in the human protein interaction network with 0.0008 density.

The performance of our method, $PMD_{pc}$, is compared with the performances of CFinder, ClusterONE, RRW, HC-PIN, and PCE-FR on the human PPI dataset derived from HPRD to validate the utilization of our method. The experimental results show that our proposed algorithm is better than the five classical approaches based on F-measure, ACC, and MMR. However, only the human PPI network was taken as the experimental dataset. The new method should be suitable for substructure detection with other sparse networks. Therefore, our algorithm will be used in the future to investigate other species of complex networks, such as gene regulatory and disease networks.

## Figures and Tables

**Table 1:** Results of six protein complexes Algorithms in HPRD Dataset.

Algorithms	Number	Precision	Recall	F-Measure	ACC	Sep	MMR	MCC
CFinder	49	0.959	0.143	0.249	0.184	0.165	0.017	0.327
ClusterONE	755	0.295	0.186	0.229	0.333	0.209	0.084	0.391
RRW	167	0.671	0.190	0.296	0.236	0.231	0.034	0.209
HC-PIN	99	0.646	0.140	0.230	0.256	0.233	0.024	0.196
PCE-FR	274	0.534	0.178	0.267	0.279	0.169	0.029	0.035
$PMD_{pc}$	118	0.451	0.356	0.398	0.362	0.777	0.010	0.343

## Supplementary Materials

The following are available online.
